# Induction of Barley Silicon Transporter *HvLsi1* and *HvLsi2*, increased silicon concentration in the shoot and regulated Starch and ABA Homeostasis under Osmotic stress and Concomitant Potassium Deficiency

**DOI:** 10.3389/fpls.2017.01359

**Published:** 2017-08-03

**Authors:** Seyed A. Hosseini, Anne Maillard, Mohammad R. Hajirezaei, Nusrat Ali, Adrian Schwarzenberg, Frank Jamois, Jean-Claude Yvin

**Affiliations:** ^1^Plant Nutrition Department, Centre Mondial de I'lnnovation Roullier Saint Malo, France; ^2^Molecular Plant Nutrition Group, Physiology and Cell Biology, Leibniz-Institute of Plant Genetics and Crop Plant Research Gatersleben, Germany

**Keywords:** barley, nutrients, silicon transporters, primary metabolites, hormones, osmotic stress

## Abstract

Drought is one of the major stress factors reducing cereal production worldwide. There is ample evidence that the mineral nutrient status of plants plays a critical role in increasing plant tolerance to different biotic and abiotic stresses. In this regard, the important role of various nutrients e.g., potassium (K) or silicon (Si) in the mitigation of different stress factors, such as drought, heat or frost has been well documented. Si application has been reported to ameliorate plant nutrient deficiency. Here, we used K and Si either solely or in combination to investigate whether an additive positive effect on barley growth can be achieved under osmotic stress and which mechanisms contribute to a better tolerance to osmotic stress. To achieve this goal, barley plants were subjected to polyethylene glycol (PEG)-induced osmotic stress under low or high K supply and two Si regimes. The results showed that barley silicon transporters *HvLsi1* and *HvLsi2* regulate the accumulation of Si in the shoot only when plant suffered from K deficiency. Si, in turn, increased the starch level under both osmotic stress and K deficiency and modulated the glycolytic and TCA pathways. Hormone profiling revealed that the beneficial effect of Si is most likely mediated also by ABA homeostasis and active cytokinin isopentenyl adenine (iP). We conclude that Si may effectively improve stress tolerance under K deficient condition in particular when additional stress like osmotic stress interferes.

## Introduction

Agricultural production is subjected to the variety of biotic and abiotic stresses that can substantially reduce the crop yield (Alegre, 2004; Avramova et al., 2016). Drought is one of the major abiotic stress reducing crop production worldwide (De Carvalho, 2008). Drought occurs over the world, even in wet and humid regions like Asia, sub-Saharan Africa, and Central and South America. Due to predicted climate change, it is expected to have often dry periods during spring and rainy summers in Northern and longer dry periods in the southern Europe (Marshall et al., 2012). According to published information in the Energy Information Administration (EIA), Unites States, Europe and Australia suffered crop losses of 20, 4.7, and 1.2 billion U.S. dollars, respectively. Therefore, improving biomass production and seed yield under suboptimal water availability or other abiotic stresses is now even more urgent (Marshall et al., 2012).

It is well described that the mineral nutrient status of plants plays a vital role in increasing plant tolerance to different biotic and abiotic stresses (Marschner, 2011). Among the mineral nutrition, K involves in many diverse processes including plasma membrane polarization, growth, regulation of stomata and uptake of other nutrients (Wang and Wu, 2013). It is also involved in the activation of enzymes, and a high K concentration is required for optimal protein synthesis and photosynthesis (Marschner, 2011). The important role of K in the mitigation of different stress factors, such as drought, heat or frost has been substantially documented (Römheld and Kirkby, 2010; Hosseini et al., 2016). This is partially due to the role of K in controlling cell turgor and stomatal closure but also to its role in stress signaling (Cheong et al., 2007; Pandey et al., 2007).

Si is an abundant mineral element in the earth's crust and still has not yet been classified as an essential mineral element (Liang et al., 2015). Over the last decade, numerous studies have considered to explore the mechanism(s) by which Si affects the agricultural and crop productivity and quality (Liang et al., 2015). In particular, the role of Si in alleviating plants from abiotic and biotic stresses in many plant species has been studied well (Liang et al., 2015). Much attention has been focussed in investigating the mechanisms by which Si mediates drought tolerance in higher plants like rice (Chen et al., 2011), wheat (Gong et al., 2005; Ma et al., 2015), and soybean (Miao et al., 2010). Si application has also been widely reported to alleviate plant nutrient deficiency (Chen et al., 2016b). Recently, it has been shown that Si ameliorated the soybean seedlings growth in the K-deficient medium by decreasing the peroxidation of lipids and oxidative stress via the modulation of antioxidant enzymes (Miao et al., 2010). In addition, Chen et al. (2016a) have shown that in sorghum, Si supply moderated K-deficiency-induced leaf chlorosis by decreasing the concentration of the polyamine putrescine (Chen et al., 2016a). Recently, it was shown in sorghum that Si alleviated K deficiency symptoms by enhancing hydraulic conductivity and improving the plant water status under K-deficient conditions (Chen et al., 2016b). The authors demonstrated two mechanisms by which Si increased the root hydraulic conductance in sorghum plants grown under K-deficient condition. In the first mechanism, Si enhances the aquaporin activity by upregulating the aquaporin genes (PIPs) or maintaining the aquaporin activity. In the second mechanism, SKOR gene (which mediates K loading into the xylem) increases the K translocation into the xylem which in turn results in increased root hydraulic conductance and higher hydraulic conductance thus contributes to an increased water uptake. Increasing water uptake and transport then reduced dehydration stress and mitigated the K deficiency. However, all previous studies have not considered the relevance of Si nutrition on metabolite and hormonal regulation.

A cooperative transporter system in the roots mediates the uptake and the accumulation of Si in different crops. In rice, *OsLsi1* belong to a noduline-26 major intrinsic protein 3 (*NIP3*) subfamily of aquaporins and *OsLsi2;* a putative anion-channel transporter, are the two primary Si transporters which contribute to Si uptake and accumulation (Ma et al., 2006, 2007; Ma and Yamaji, 2015). The homolog of both *OsLsi1* and *OsLsi2* has also been identified in barley (Chiba et al., 2009; Mitani et al., 2009) and other crops like maize and wheat (Mitani et al., 2009; Montpetit et al., 2012). It was shown that the expression of these two genes was down-regulated in rice upon Si supply (Ma et al., 2006, 2007), in response to drought stress (Yamaji and Ma, 2007) and with ABA treatment (Yamaji and Ma, 2011). In barley, the expression of *HvLsi1* was unchanged by Si application (Chiba et al., 2009), while *HvLsi2* expression was down-regulated (Mitani et al., 2009). Recently, Mitani-Ueno et al. (2016) found that the Si accumulation in the shoot of rice plant controlled the expression of *OsLsi1* and *OsLsi2*. However, the expression pattern of these two genes and related mechanisms responsible for Si accumulation under drought, K deficiency or either has not yet been investigated.

In order to investigate the role of Si nutrition under osmotic stress and concomitant K deficiency, barley plants were exposed to polyethylene glycol (PEG)-induced osmotic stress which partly mimics drought stress under low or high K and two Si regimes. In particular, the expression of barley Si transporters genes and the mechanisms contributing to a better osmotic stress tolerance have been investigated in more detail. We could demonstrate that the regulation of *HvLsi1* and *HvLsi2* controlled the accumulation of Si in the shoot only when plant suffered from K deficiency. Further, higher Si accumulation in the shoot of barley plants resulted in an increase of chlorophyll level and modulated starch synthesis and phytohormones (ABA, CKs) under osmotic stress and K deficiency, which might contribute to better stress tolerance.

## Materials and methods

### Plant materials and growth conditions

Seeds of *Hordeum vulgare* cv. Irina was germinated on vermiculite for three days in the dark and additional four days under light conditions. After one week, seedlings were transplanted to a 5.9 L tank in a growth chamber that was set to a 14/10 h day/night cycle at a day/night temperature of 28/25°C with 40–50% relative humidity. Plant were divided into two batches: (i) K-sufficient plants were grown with a complete solution: Ca(NO_3_) 2 mM, K_2_SO_4_ 1 mM, MgSO_4_ 0.5 mM, NH_4_H_2_PO_4_ 0.5 mM, CaCl_2_ 0.5 mM, H_3_BO_3_ 0.001 mM, MnSO_4_ 0.0025 mM, ZnSO_4_ 0.0005 mM, CuSO_4_ 0.0002 mM, (NH_4_)_6_Mo_7_O_24_ 0.00001 mM and EDTA, 2NaFe 0.1 mM while (ii) K-deficient plants were grown in the same solution with a low concentration of K_2_SO_4_ 0.05 mM. The nutrient solution was buffered to pH 5.9 and renewed every 2 days and continuously aerated. After 1 week of K-deficiency, two concentrations of Si (0.5 and 1 mM) were introduced to the culture solution by the addition of Na_2_SiO_3_. To maintain the same concentration of Na, 2 mM and 1 mM of NaCl was added to the nutrient solution with no Si supply and with 0.5 mM of Na_2_SiO_3_ supply, respectively. Two weeks after the application of K, drought conditions were simulated by the addition of PEG-6000 at 190 g L^−1^ in the nutrient solution to achieve osmotic stress levels of approximately −0.5 MPa for the duration of 5 days. Another batch of plants were kept under control condition without supplying PEG into culture solution. Three independent samples, each consisting of six plants, were harvested 17 days after transplanting for each treatment. The last fully expanded leaves were harvested and immediately frozen in liquid nitrogen and stored at −80°C until further molecular and biochemical analysis. An aliquot of each tissue was weighed, dried in an oven at 60°C, weighed for dry matter determination and ground with Inox beads in an oscillating grinder (Mixer Mill MM400; Retsch, Haan, Germany) for elementary analysis. In parallel, root samples were harvested for determination of elements and both fresh and dry weights.

### Chlorophyll measurement

For estimation of chlorophyll levels, spectral plant analysis diagnostic (DUALEX) readings were taken before the final harvest using a DUALEX photometer (DUALEX v4.5, Force A, France). Measurements were taken from the middle region of 4 fully expanded leaves (three times per leaf) and mean values were recorded for each treatment.

### Element analysis

For determination of elements, fully expanded leaves were dried for 48 h at 65°C (Multiwave PRO, Anton Paar) and digested using 8 mL of concentrated HNO_3_ (65% Merck). For Si determination, 8 ml of 0.1 M Tiron solution buffered at pH 10.5 were added to 25 mg of DW which was continuously shaken for at least 4 h at 65°C in a shaker incubator (Infors HT, Minitron). After cooling, 7 mL of H_2_O_2_ (Roth) was added to destroy Tiron. The tubes were shaken horizontally in a water bath at 85°C until the solution turned colorless. The samples were then centrifuged at 4,000 rpm at 25°C for 10 min before analysis. The elements were analyzed by Inductively Coupled Plasma Optical Emission Spectrometry (iCAP 6500 dual OES spectrometer, Thermo Scientific) by using Yttrium solution (1 ppm, Merck) as an internal standard.

### Determination of primary metabolites

Soluble sugars and starch were determined in fully expanded leaves according to Kim et al. (2013). 50 mg frozen flag leaf material were homogenized in liquid nitrogen, dissolved in 0.75 ml of 80% (v/v) ethanol and incubated at 80°C for 60 min. Crude extracts were centrifuged at 14,000 rpm for 5 min at 4°C, and the upper phase was concentrated in a speed vacuum concentrator (Christ, Germany) at 45°C for 180 min. The pellet was resuspended in 0.25 ml HPLC-grade water and shaken for 15 min at 4°C. Hexokinase (HK), phosphoglucoisomerase (PGI) and β-fructosidase were added successively to measure Glc, Fru and Suc as described in Kim et al. (2013). The residue of sugar extraction was washed twice with 1 ml of 80% (v/v) ethanol. Starch was decomposed with 0.4 ml of 0.2 N KOH for 16 h at 4°C and neutralized with 70 μl of 1 M acetic acid. Hydrolysis of starch was performed using a 1:1 ratio of sample and a buffer containing 50 mM sodium acetate, pH 5.2 and 7 units mg^−1^ of amyloglucosidase (Roche, Germany). The cocktail was incubated for 16 h at 37°C. Determination of produced Glc was performed according to Kim et al. (2013).

Free amino acids were extracted and determined as described by Höller et al. (2014). Analysis and quantification of metabolites were performed essentially as described by Hosseini et al. (2016).

### Phytohormone measurements

Acidic phytohormones, ABA, PA, DPA, SA, and IAA were analyzed by a UHPLC-MS/MS system. The separation and detection were achieved using a Nexera X2 UHPLC system (Shimadzu, Japan) coupled to a QTrap 6500+ mass spectrometer (Sciex, Canada) equipped with an IonDrive™ turbo V electrospray (ESI) source. Phytohormone separation was carried out by injecting 2 μL sample into a Kinetex Evo C18 core-shell column (100 × 2.1 mm, 2.6 μm, Phenomenex, USA), at a flow rate of 0.7 mL/min, and the column temperature was maintained at 40°C. The mobile phases were composed of solvent A Milli-Q water (18 Ω, Millipore, USA) containing 0.1% formic acid (LCMS grade, Fluka analytics, Germany), and solvent B acetonitrile LCMS grade (Fisher Optima, UK) containing 0.1% formic acid. The gradient elution started with 1% B, 0.0–5.0 min 60% B, 5.0–5.5 min 100% B, 5.5–7.0 min 100% B, 7.0-7.5 min 1% B, and 7.5–9.5 min 1% B. The ionization voltage was set to 5 kV for positive mode (auxins) and −4.5 kV for negative mode (ABA and derivates) producing mainly [M+H]^+^ and [M−H]^−^ ions, respectively. The analysis was performed in scheduled MRM mode in positive and negative mode simultaneously with a polarity switching of 5 ms.

Cytokinins separation was carried out using the same parameters as described above. The gradient elution started with 2% B, 0.0–3.0 min 20% B, 3.0–4.0 min 25% B, 4.0–4.5 min 100 % B, 4.5−6.0 min 100% B, 6.0−6.5 min 2% and 6.5−8.5 min 2% B. The capillary voltage was set to 5 kV for positive mode producing mainly [M+H]^+^ ions. All quantitative data were processed using MultiQuant software V 3.0.2 (Sciex, Canada).

### RNA isolation, cDNA synthesis, and gene expression analysis

Total RNA was extracted from 100 mg leaf or root material using Nucleospin® 8 RNA kit following the manufacturer's protocol (Macherey-Nagel). The quality of RNA was checked in 4200 Tapestation (Agilent Technologies), followed by cDNA synthesis using iScript™ Reverse Transcription Supermix (Bio-Rad). The cDNA samples were used to study gene expression by quantitative real-time PCR (CFX384 Touch™, Bio-Rad) using SsoAdvanced Universal SYBR Green Supermix (Bio-Rad). The thermal cycling protocol included a single polymerase activation step at 98°C for 3 min followed by 40 amplification cycles, a final extension step at 72°C for 5 min as well as melt-curve analysis. Each amplification cycle comprised a denaturation step at 98°C for 15 s, a primer annealing step at 60/61°C for 30 s and a brief extension at 72°C for 15 s. Primer efficiency was determined by performing standard curve analysis. All qPCR expression data were obtained from CFX Manager Software Version 3.1 (Bio-Rad). The expression of candidate genes was normalized against *GAPDH, Cyclophilin* (*CYC*), *ACTIN* and *ADP-ribosylation* factor (*ADP-RF1*). Primers for all candidate genes were designed using Primer3 software and listed in Supplemental Table 1.

### Statistical analysis

For each measurement, hydroponic experiments on *H. vulgare* were conducted with three independent biological replicates constituted of six individual plants. Data are presented as mean ± standard error (SE) for *n* = 3. All data were analyzed by SNK's test (R software) and marked by different letters when significantly different (*P* < 0.05).

## Results

### Si application enhanced chlorophyll level and delayed osmotic stress-induced leaf senescence

**Figure 1:**
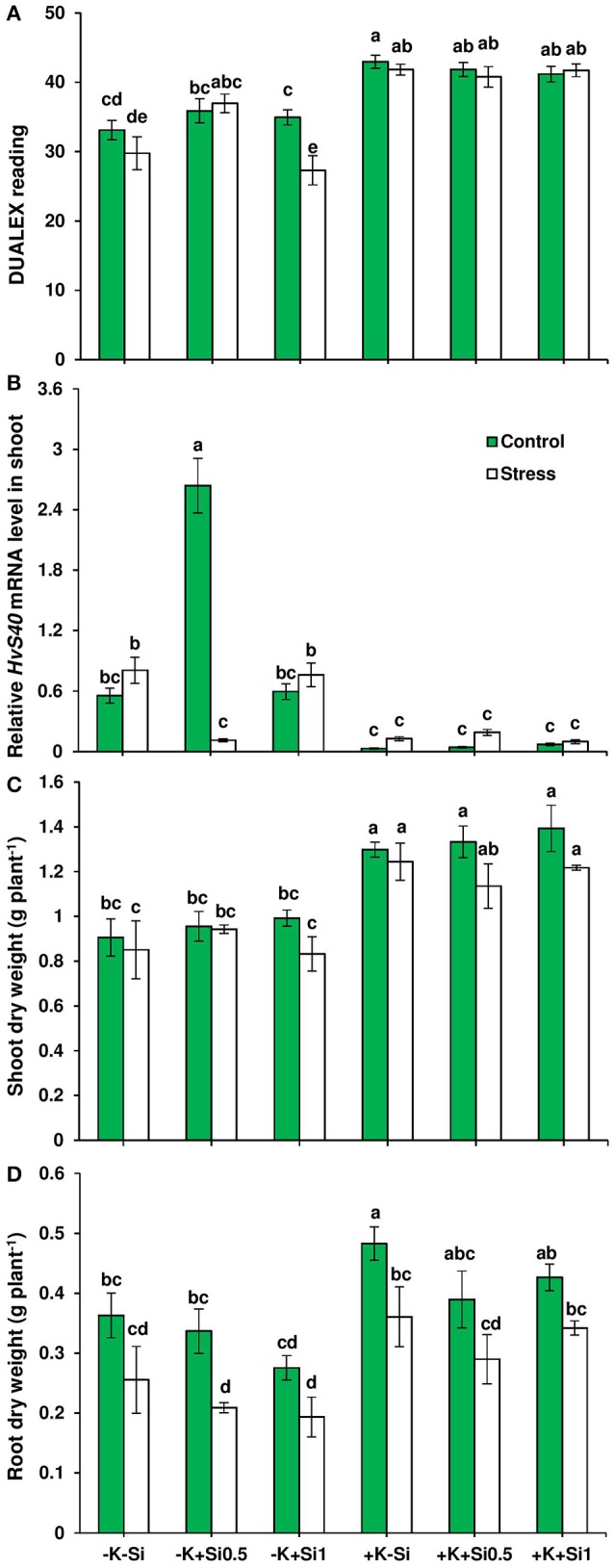
Influence of Si supply on chlorophyll level, root and shoot dry weights and expression of senescence marker gene in barley under osmotic stress. **(A)** Chlorophyll level, **(B)** relative *HvS40* mRNA levels, **(C)** shoot dry weight, and **(D)** root dry weight in barley. Plants were grown in hydroponic culture under low K (0.05 mM), high K (1 mM) and two concentrations of Si (0.5 and 1 mM). Fully expanded leaves from 25-days old plants were harvested 5 days after imposition of osmotic stress. Osmotic stress applied by polyethylene glycol [PEG 6000, 19% (w/v)] to achieve osmotic stress levels of approximately −0.5 MPa. Chlorophyll was measured by DUALEX device. Bars indicate means ± SE. Different letters denote significant differences according to SNK test (*p* < 0.05; *n* = 3).

To investigate the impact of Si nutrition on plant growth under drought and K deficiency, we grew barley plants (variety Irina) in hydroponic condition under two K regimes (0.05 mM as low K and 1 mM as high K) and two concentrations of Si (0.5 mM and 1 mM). After three weeks, plants were exposed to osmotic stress (see Materials and Methods, polyethylene glycol applied to mimic drought stress) for the duration of 5 days where the symptoms of osmotic stress in the whole plant can be visibly monitored. To assess whether chlorophyll level was modulated by Si nutritional status under osmotic stress and K deficiency, we determined chlorophyll level in fully expanded leaves. Under ample K supply, no significant changes were observed in the dualex readings, which suggests that chlorophyll level did not change upon Si application in both control and osmotic-stressed plants. Interestingly, under osmotic stress and concomitant low K supply, when Si was applied at lower (0.5 mM) concentration, a significant increase of 24% was recorded in the dualex readings, indicating higher chlorophyll level in comparison to non-Si-treated plants (Figure 1A). To further confirm the observed increase in chlorophyll level by Si supply, we examined the expression levels of *HvS40* gene, a senescence marker in barley (Krupinska et al., 2002). The expression level of this gene was suppressed under ample K supply despite osmotic stress or Si supply, while under the K-deficient condition, *HvS40* expression was induced both in non-Si-treated plants and under the higher concentration of Si (Figure 1B). Interestingly, osmotic stress and lower concentration of Si actively suppressed the expression levels of *HvS40* gene under K deficiency which coincides with higher chlorophyll levels in the same treatments (Figures 1A,B).

Next, we measured both root and shoot dry weights. Application of Si did not affect root dry weights (Figure 1D). The same trend was also observed for shoot dry weight, however, the shoot dry weight tended to slightly increase (11 %) in the presence of low Si concentration (0.5 mM) (Figure 1C). These results showed that Si application delayed osmotic stress-induced leaf senescence in response to K deficiency.

### Shoot Si concentration and expression of Si transporter genes positively correlate under both osmotic stress and K deprivation condition

**Figure 2:**
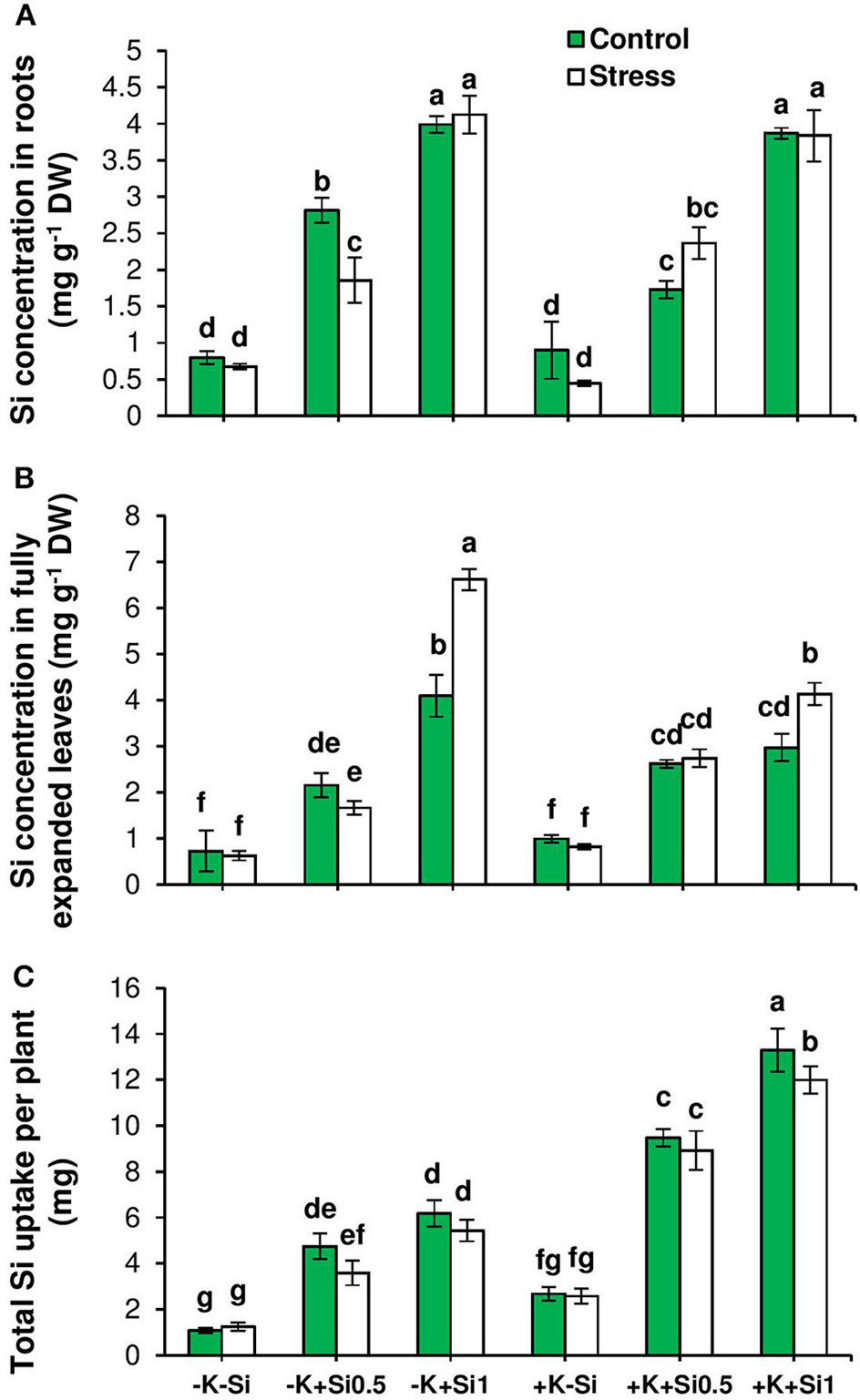
Influence of Si supply on Si concentration and total Si uptake in fully expanded leaves and roots of barley under osmotic stress. **(A)** Si concentration in root, **(B)** Si concentration in fully expanded leaves and **(C)** total Si uptake in barley. Plants were grown in hydroponic culture under low K (0.05 mM), high K (1 mM) and two concentrations of Si (0.5 and 1 mM). Fully expanded leaves from 25-days old plants were harvested 5 days after imposition of osmotic stress. Osmotic stress applied by polyethylene glycol [PEG 6000, 19% (w/v)] to achieve osmotic stress levels of approximately −0.5 MPa. Bars indicate means ± SE. Different letters denote significant differences according to SNK test (*p* < 0.05; *n* = 3).

The response of barley Si transporters to osmotic stress and K deficiency was analyzed by evaluating the expression pattern of *HvLsi1, HvLsi2*, and *HvLsi6*. In parallel, we determined the Si concentration in both roots and shoots. Irrespective of K level in the medium, Si concentration increased in roots upon Si application under both control and osmotic stress conditions (Figure 2A). The same trend was observed for Si concentration in the leaves. The increase in Si concentration in leaves was significantly higher (up to 60%) in K-deficient plants in comparison to K-sufficient plants when Si was added in higher concentration (Figure 2B) in particular when plants were exposed to osmotic stress (Figure 2C).

**Figure 3:**
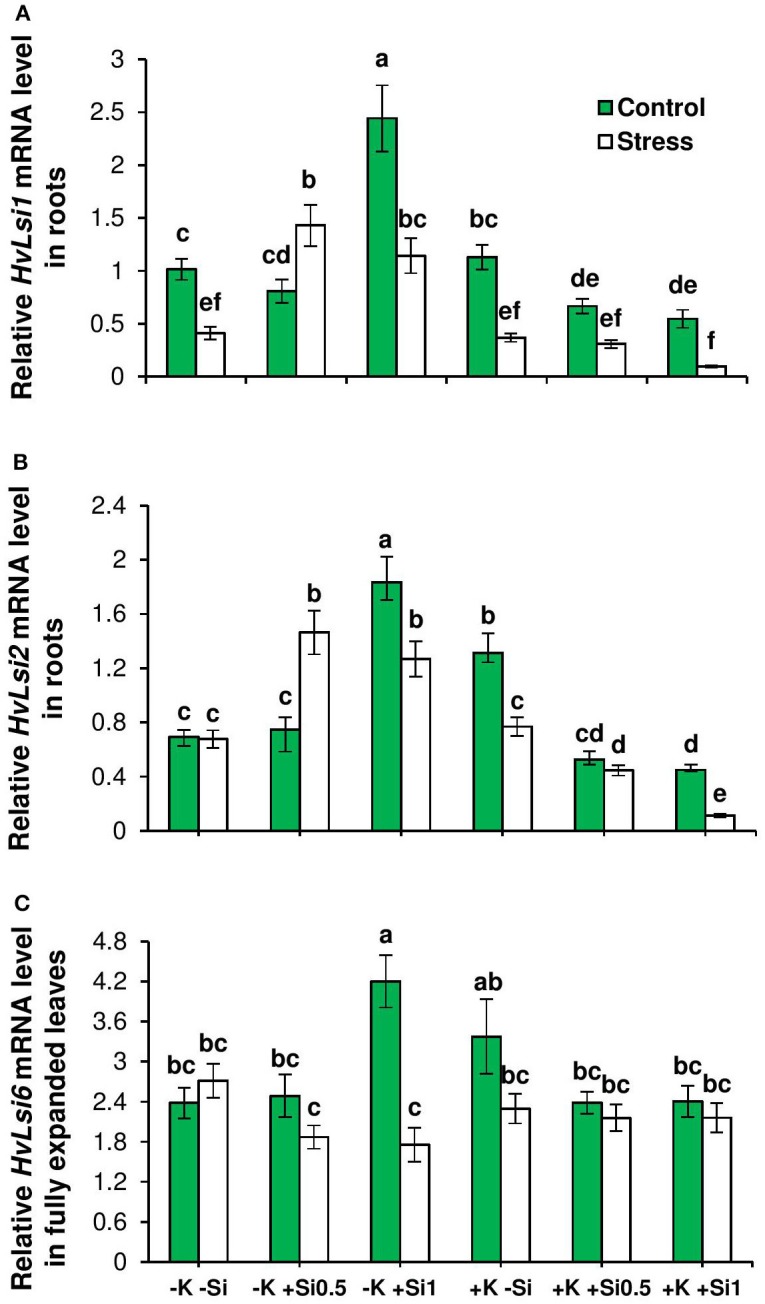
Influence of Si supply on the expression of genes involved in Si transporters in fully expanded leaves and roots of barley under osmotic stress. **(A)** Relative *HvLsi1* mRNA levels, **(B)** relative *HvLsi2* mRNA levels, and **(C)** relative *HvLsi6* mRNA levels in fully expanded leaves and root of barley. Plants were grown in hydroponic culture under low K (0.05 mM), high K (1 mM) and two concentrations of Si (0.5 and 1 mM). Fully expanded leaves from 25-days old plants were harvested 5 days after imposition of osmotic stress. Osmotic stress applied by polyethylene glycol [PEG 6000, 19% (w/v)] to achieve osmotic stress levels of approximately −0.5 MPa. Bars indicate means ± SE. Different letters denote significant differences according to SNK test (*p* < 0.05; *n* = 3).

Further, the expression levels of *HvLsi1* and *HvLsi2* genes contributing to Si uptake in barley roots were analyzed (Chiba et al., 2009). Transcript levels of both genes were significantly up-regulated under osmotic stress and concomitant K deficiency by Si supply in contrast to those with sufficient K in the medium in which the genes were down-regulated (Figures 3A,B). In addition, the transcript abundance of *HvLsi6* was analyzed. Expression pattern of this gene showed a reduction when plants were subjected to osmotic stress and K deficiency. By contrast and under control condition, the transcript level of this gene was up-regulated upon application of Si when plants suffered from K deficiency (Figure 3C). No consistent responses to Si supply under K-sufficient condition either under control or osmotic stress could be observed (Figure 3C).

**Figure 4:**
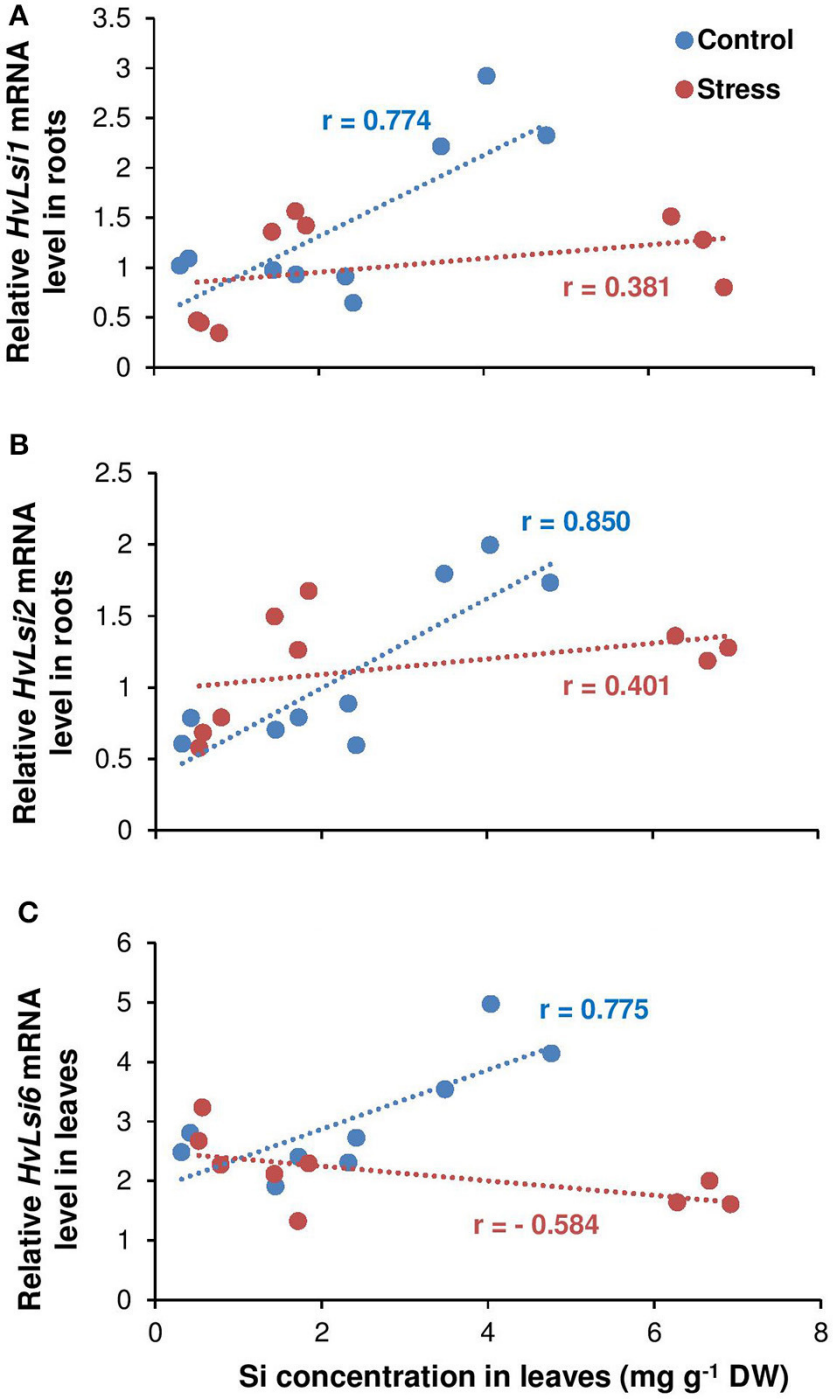
Correlation between Si concentrations in leaves and the expression of Si transporters in barley under osmotic stress. Correlation between Si concentrations in shoot and expression of **(A)** *HvLsi1*, **(B)** *HvLsi2*, and **(C)** *HvLsi6* genes under K deficient condition (red under control and blue under osmotic stress). Plants were grown in hydroponic culture under low K (0.05 mM), high K (1 mM) and two concentrations of Si (0.5 and 1 mM). Fully expanded leaves from 25-days old plants were harvested 5 days after imposition of osmotic stress. Osmotic stress applied by polyethylene glycol [PEG 6000, 19% (w/v)] to achieve osmotic stress levels of approximately −0.5 MPa.

**Figure 5:**
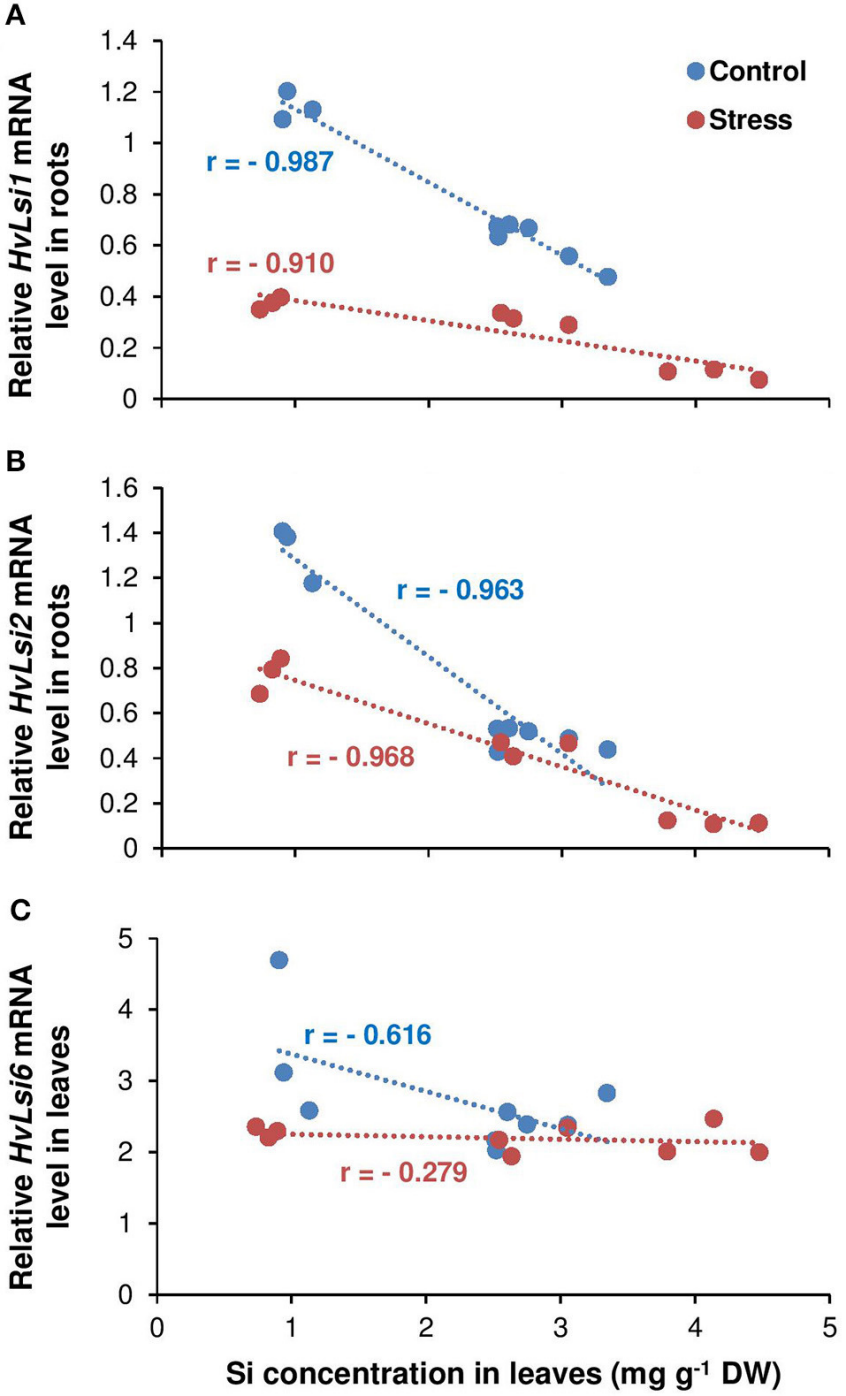
Correlation between Si concentrations in leaves and the expression of Si transporters in barley under osmotic stress. Correlation between Si concentrations in shoot and expression of **(A)** *HvLsi1*, **(B)** *HvLsi2*, and **(C)** *HvLsi6* genes under K-sufficient condition (red under control and blue under stress). Plants were grown in hydroponic culture under low K (0.05 mM), high K (1 mM) and two concentrations of Si (0.5 and 1 mM). Fully expanded leaves from 25-days old plants were harvested 5 days after imposition of osmotic stress. Osmotic stress applied by polyethylene glycol [PEG 6000, 19% (w/v)] to achieve osmotic stress levels of approximately −0.5 MPa.

To find the relationship between shoot Si and the expression of Si transporter genes, a correlation analysis was performed between shoot Si and the expression levels of *HvLsi1, HvLsi2*, and *HvLsi6* genes in roots and fully expanded leaves. The results showed a positive correlation between shoot Si concentration and the expression levels of both *HvLsi1* and *HvLsi2* (*r* = 0.77 and *r* = 0.85 for *HvLsi1* and *HvLsi2*, respectively under control and *r* = 0.38 and *r* = 0.40 for *HvLsi1* and *HvLsi2*, respectively under osmotic stress) only when plants were exposed to K deficiency (Figures 4A,B). In contrast, under sufficient K supply this correlation was strongly negative (*r* = −0.99 and *r* = −0.96 for *HvLsi1* and *HvLsi2*, respectively under control and *r* = −0.91 and *r* = −0.97 for *HvLsi1* and *HvLsi2*, respectively under osmotic stress; Figures 5A,B). These results suggest that the expression of *HvLsi1* and *HvLsi2* genes in roots positively correlate with the Si accumulation in the shoot.

The correlation analysis also revealed a positive relation (*r* = 0.78) between Si concentration in leaves and the expression of *HvLsi6* under control and limiting K conditions. This correlation was negative (*r* = −0.58) when plants were subjected to osmotic stress (Figure 4C). Under sufficient K supply, the correlation between all three Si transporter genes and Si concentration was negative in shoots under both control and osmotic stress condition (Figures 5A–C) indicating that *HvLsi6* contributes to Si accumulation in shoots only under K deficiency and control condition.

### Si application reduced the level of ABA in shoot under osmotic stress and concomitant K deficiency

**Figure 6:**
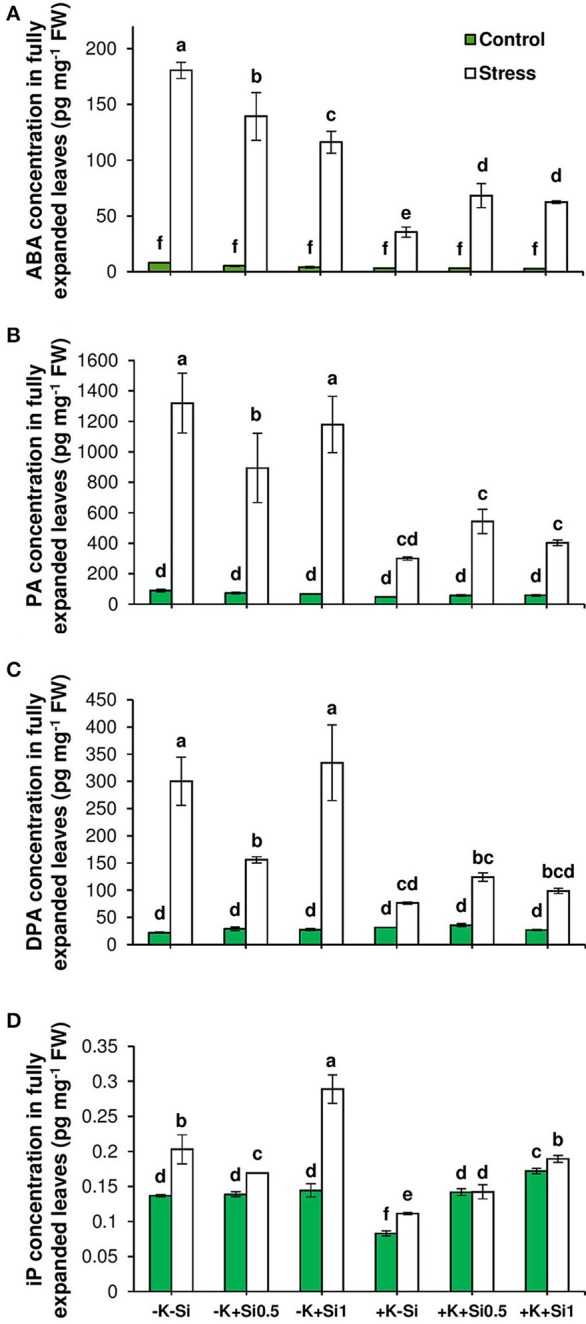
Influence of Si supply on concentrations of abscisic acid (ABA), ABA degradation products phaseic acid (PA) and diphaseic acid (DPA) and isopentenyl adenine (iP) in fully expanded leaves of barley under osmotic stress. **(A)** ABA, **(B)** PA **(C)** DPA and **(D)** iP concentrations in fully expanded leaves of barley. Plants were grown in hydroponic culture under low K (0.05 mM), high K (1 mM) and two concentrations of Si (0.5 and 1 mM). Fully expanded leaves from 25-days old plants were harvested 5 days after imposition of osmotic stress. Osmotic stress applied by polyethylene glycol [PEG 6000, 19% (w/v)] to achieve drought stress levels of approximately −0.5 MPa. Bars indicate means ± SE. Different letters denote significant differences according to SNK test (*p* < 0.05; *n* = 3).

Phytohormone profiling revealed that the concentration of abscisic acid (ABA) as an indicator of drought stress, increased under osmotic stress despite the K level in the medium and even higher under K deficiency. Unlike K sufficient condition, ABA concentration was reduced in K-deficient plants by Si application and this reduction was even pronounced at the concentration of 1 mM Si (Figure 6A). The same trend was observed for ABA degradation products phaseic acid (PA) and dehydro-phaseic acid (DPA). However, plants receiving a higher concentration of Si (1 mM) were able to accumulate higher PA and DPA under osmotic stress and concomitant K deficiency (Figures 6B,C). This emphasizes that lower ABA level is associated with a higher turnover of ABA when Si concentration increases in the medium.

**Figure 7:**
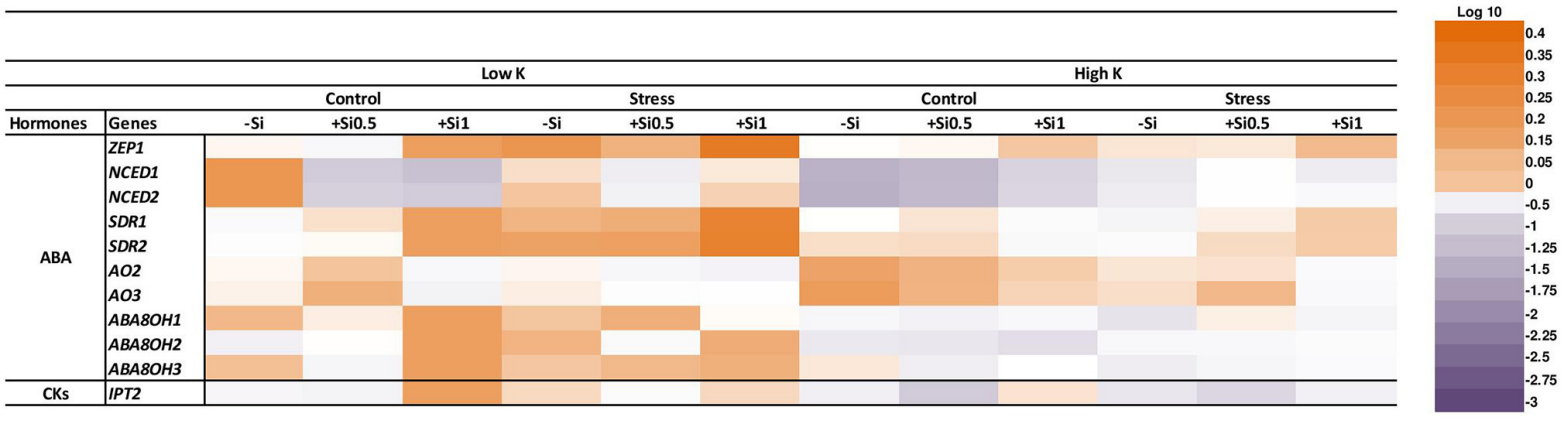
Influence of Si supply on the expression of genes involved in ABA, CKs biosynthesis and ABA degradation in fully expanded leaves of barley under osmotic stress. Fully expanded leaves from 25-days old plants were harvested 5 days after imposition of osmotic stress. Osmotic stress applied by polyethylene glycol [PEG 6000, 19% (w/v)] to achieve drought stress levels of approximately −0.5 MPa. Purple color indicates a decrease and orange indicates an increase in gene expression levels of different hormonal pathways. Different shades of purple and orange express the extent of the change according to the color bar provided (log10). White indicates no change.

To identify the mechanism of Si nutrition on ABA biosynthesis and degradation, we studied the expression of plastid-located ABA biosynthetic genes, zeaxanthin epoxidase (*HvZEP1*), which catalyzes the first step of ABA biosynthesis and also the expression levels of two 9-cis-epoxycarotenoid dioxygenase genes (*HvNCED1* and *HvNCED2*) which regulates the key steps of ABA biosynthesis (Nishiyama et al., 2011). Expression of *HvZEP1* was up-regulated under osmotic stress and K deficiency. However, compared to non-Si-treated plants, the expression of *HvZEP1* initially decreased at low concentration of Si and increased 2-fold when the Si concentration was high in the medium (Figure 7). There was no consistent change in the expression of *HvZEP1* gene under K-sufficient condition. The result of the quantitative qRT-PCR analysis indicated that the expression level of both *HvNCED1* and *HvNCED2* were induced under osmotic stress in spite of the K level in the medium. The expression level of both genes decreased at low concentration of Si and restored to the same level as in non-treated plants when Si was applied in higher concentration (Figure 7). Monitoring the transcript levels of genes coding for short-chain dehydrogenase/reductase (*HvSDR1* and *HvSDR2*) which catalyzes the conversion of intermediate product xanthoxin to abscisic aldehyde (Seiler et al., 2011) resulted in an increase of both genes under osmotic stress condition. This increase was significantly higher with increasing Si concentration (Figure 7). The same trend was observed under control and K deficiency for the expression level of *HvSDR1* gene in both Si-treated plants compared to plants without Si. The transcript level of *HvSDR2* gene significantly increased under control and K-deficient conditions in the presence of high Si concentration (Figure 7). We also examined the expression levels of two genes coding for aldehyde oxidases, *HvAO2* and *HvAO3* which catalyze the last step in ABA biosynthesis by converting ABA aldehyde to ABA (Seiler et al., 2011). Under osmotic stress and concomitant K deficiency, the expression level of both genes did not differ; however; under control condition and low supply of Si, the expression of both genes were significantly up-regulated (Figure 7). Under sufficient K supply and control condition, the expression of both genes was down-regulated upon Si supply while the expression of *HvAO3* gene was up-regulated under osmotic stress and low concentration of Si (Figure 7).

In addition, we also analyzed the expression of ABA catabolic genes *HvABA8'OH-1, HvABA8*′*OH-2*, and *HvABA8*′*OH-3*. Out of 3 genes, only the expression of *HvABA8*′*OH-1* and *HvABA8*′*OH-3* genes was induced by low concentration of Si under osmotic stress and concomitant K deficiency (Figure 7).

### Si application increased the level of active cytokinin iP under osmotic stress and K deficiency

Cytokinins (CKs) are thought to play a major role in abiotic stress responses (Zwack and Rashotte, 2015). Therefore, different CK forms were analyzed in this study. Interestingly, the concentration of iP as active CKs was increased in response to osmotic stress and K deficiency followed by a decline upon application of 0.5 mM Si and restored again to the same level in non-treated Si plants when the Si concentration increased to 1 mM (Figure 6D). The analysis of the expression level of isopentenyl transferase gene (*HvIPT2*) that encodes a rate-limiting enzyme in cytokinin biosynthesis indicated a significant correlation between the transcript level and the hormone level. (Figures 6, 7). This gene responded to Si in a dose-dependent manner as it was reduced with low concentration of Si under osmotic stress and K deficiency and increased with increasing Si concentration even under control condition (Figure 7).

Concentrations of phytohormones SA, IAA, GA3 and ethylene and the respective genes involved in their biosynthesis/signaling pathways were also determined. However, we did not observe any consistent change either at metabolite or transcript level of these phytohormones (Supplemental Figure 1).

### Metabolite changes in roots and shoots upon Si application

The effect of K and Si on primary metabolism was analyzed through metabolite profiling, which included carbohydrates and organic acids in roots and shoots. The metabolite profiles revealed a significant effect of Si supply on the levels of primary metabolites under osmotic stress and concomitant K deficiency. Sucrose (Suc) level increased significantly in root under osmotic stress irrespective of the K or Si treatments and no changes in glucose (Glu) and fructose (Fru) levels could be observed in either treatments (Table 1). Among detected organic acids in the roots, the level of malate, fumarate and citrate increased under osmotic stress and K-deficient conditions in the presence of Si. In addition, the levels of the same organic acids increased under osmotic stress and sufficient K supply irrespective of the Si concentration in the medium. The levels of phosphoenolpyruvate (PEP) and 3-phosphoglycerate (3PGA), involved in the glycolysis pathway increased under osmotic stress and concomitant K deficiency (Table 1).

**Table 1:** Influence of Si supply on metabolite concentrations in roots of barley under Osmotic stress.

		**Low K**						**High K**					
		**Control**			**Stress**			**Control**			**Stress**		
**Pathways**	**Metabolite**	**−K-Si**	**−K Si0.5**	**−K Si1**	**−K-Si**	**−K Si0.5**	**−K Si1**	**−K-Si**	**−K Si0.5**	**−K Si1**	**+K-Si**	**+K Si0.5**	**+K Si1**
Sugars	Glucose	6.7 a	3.2 b	2.9 b	2.8 b	3.0 b	1.9 b	1.7 b	1.8 b	2.1 b	1.7 b	1.6 b	1.5 b
	Fructose	0.01 a	0.005 b	0.004 b	0.002 b	0.006 b	0.002 b	0.002 b	0.003 b	0.003 b	0.002 b	0.002 b	0.002 b
	Sucrose	2.4 cd	2.1 cd	2.3 cd	3.5 bcd	5.4 a	5.1 ab	1.6 d	2.0 cd	3.1 bcd	4.4 ab	4.5 ab	3.8 abc
Glycolysis	3PGA	36.6 ab	32.4 ab	35.6 ab	12.9 b	40.0 a	18.6 ab	29.7 ab	27.2 ab	23.7 ab	30.5 ab	18.3 ab	25.5 ab
	PEP	10.8 a	5.3 cde	9.7 ab	2.4 e	7.2 bcd	2.8 e	5.0 cde	4.5 cde	3.1 e	8.0 abc	4.0 de	5.0 cde
TCA cycle	Fumarate	4130.2 a	3030 cd	3576 a	1189 d	3339 cd	3441 ab	1256 cd	1364 bc	2195 bc	3152 bc	3219 bc	3237 bc
	Citrate	437 bcd	758 bcd	632 bcd	132 d	1331 a	812 abc	272.0 cd	294.6 cd	368.2 cd	1033 ab	734 bcd	1036 ab
	Malate	843 ab	857 ab	739 ab	222 b	1150 ab	960.9 ab	778 ab	908 ab	879 ab	1253 ab	1444 a	1750 a

*Concentrations of soluble sugars, amino acids and primary metabolites were measured in roots of barley. Plants were grown in hydroponic culture under low K (0.05 Mm), high K (1 mM) and two concentrations of Si (0.5 and 1 mM). Roots from 25-days old plants were harvested 5 days after imposition of osmotic stress. Osmotic stress applied by polyethylene glycol [PEG 6000, 19% (w/v)] to achieve osmotic stress levels of approximately −0.5 Mpa. Bars indicate means ± SE. Different letters denote significant differences according to SNK test (p < 0.05; n = 3). The concentration of metabolites calculated based on nmol/g FW. 3PGA, 3 phosphoglycerate; PEP, phosphoenolpyruvate*.

In shoots, the soluble sugars Glu and Suc did not change within the treatments and only Fru level increased under osmotic stress and K-deficient conditions and was significantly higher in non-Si-treated plants. A definite change was observed in the level of starch, which significantly increased under osmotic stress and K deficiency in plants that were supplied with Si (Table 2). The same trend in the starch increase was also observed under sufficient K supply both under control and osmotic stress conditions. Among glycolytic metabolites, the level of sucrose-6-P, 3PGA and PEP and among the TCA cycle metabolites the levels of fumarate, citrate and isocitrate were changed (Table 2). The level of 3PGA and isocitrate increased by application of Si in both control and osmotic stress conditions when K was less in the medium. In addition, the level of fumarate and malate increased under osmotic stress and K deficiency in both non-Si-treated and low Si-treated plants. Sucrose-6-P concentration increased only under osmotic stress and concomitant K deficiency. Under sufficient K supply, the levels of malate, citrate and isocitrate increased for both control and osmotic stressed plants. We also observed a marked increase in PEP and 3PGA levels under control conditions when plants received a low concentration of Si and sufficient K (Table 2).

**Table 2:** Influence of Si supply on metabolite concentrations in fully expanded leaves of barley under Osmotic stress.

		**Low K**						**High K**					
		**Control**			**Stress**			**Control**			**Stress**		
**Pathways**	**Metabolite**	**−K-Si**	**−K Si0.5**	**−K Si1**	**−K-Si**	**−K Si0.5**	**−K Si1**	**−K-Si**	**−K Si0.5**	**−K Si1**	**+K-Si**	**+K Si0.5**	**+K Si1**
Sugars	Glucose	33.1 a	12.8 bc	10.6 bcd	18.2 b	17.7 b	14.1 bc	2.6 d	4.8 cd	6.4 cd	7.5 cd	9.8 bcd	8.1 cd
	Fructose	0.009 b	0.004 cd	0.004 cd	0.013 a	0.009 b	0.010 b	0.002 d	0.003 cd	0.004 cd	0.004 cd	0.006 c	0.005 c
	Sucrose	23.4 ab	25.4 ab	30.1 a	22.2 ab	27.7 a	28.3 a	17.7 b	27.3 a	30.4 a	27.7 a	31.1 a	31.9 a
	Starch	6.29 a	5.08 abc	5.83 ab	1.47 e	4.4 abcd	6.57 a	2.48 de	4.66 abcd	3.76 bcd	3.13 cde	4.71 abcd	5.35 abc
Starch metabolism	Sucrose-6-P	0.274 b	0.241 b	0.196 b	0.324 b	0.675 a	0.290 b	0.139 b	0.228 b	0.304 b	0.407 b	0.397 b	0.331 b
Glycolysis	3PGA	23.7 g	65.2 cdef	70 bcdef	18.5 g	87 bcdef	44 efg	106 b	247 a	96 b	75 bcde	52 cdefg	33.4 fg
	PEP	3.0 d	9.1 bc	8.1 bc	2.5 d	9.4 bc	3.2 d	11.2 b	25.3 a	9.9 b	8.4 bc	4.0 cd	2.3 d
TCA cycle	Fumarate	11062 e	24237cde	25386 de	38640 b	56469 a	27669 bc	14446 de	17881 cd	19272 cd	19412 cd	30740 bc	37223 b
	Citrate	345.8 d	11011 ab	16240 ab	10805 bc	8408 bc	7966 bc	6577 bc	12530 ab	11757 ab	9774 ab	12681 ab	14361 ab
	Isocitrate	122 c	315 c	690 bc	1005 bc	1686 b	1427 bc	1042 bc	1800 b	1790 b	3034 a	2867 a	3071 a
	Malate	11564 bc	11757 bc	14134 bc	21415 ab	22208 ab	14439 bc	14397 abc	22727 ab	25436 a	18878 ab	23153 ab	26390 a

*Concentrations of soluble and insoluble sugars, amino acids and primary metabolites were measured in fully expanded leaves of barley. Plants were grown in hydroponic culture under low K (0.05 Mm), high K (1 mM) and two concentrations of Si (0.5 and 1 mM). Fully expanded leaves from 25-days old plants were harvested 5 days after imposition of osmotic stress. Osmotic stress was applied by polyethylene glycol [PEG 6000, 19% (w/v)] to achieve osmotic stress levels of approximately −0.5 MPa. Different letters denote significant differences according to SNK test (p < 0.05; n = 3). The concentration of metabolites calculated based on nmol/g FW. Suc6P, sucros-6-phosphate, 3PGA, 3 phosphoglycerate*.

## Discussion

Besides the prominent role of K in mitigating drought stress, Si has also been shown to alleviate drought stress responses in many crop plants (Gong et al., 2005; Gunes et al., 2008; Chen et al., 2011; Ma et al., 2015). Most previous studies have investigated the beneficial effects of Si in response to a single stress condition. Few other studies demonstrated the role of Si nutrition under K deficiency. To date, no evidence is available for an interaction between K and Si nutrition, in particular when additional abiotic stress like osmotic stress interferes. Therefore, we studied the cumulative effect of different nutrients in combination with osmotic stress to address the question whether barley growth can be improved under K deficiency and osmotic stress by the application of Si. We could demonstrate that application of Si to barley plants suffering from osmotic stress and K deficiency induced Si transporters resulting in translocation of Si to the shoot organ that increased chlorophyll and starch level and regulated ABA homeostasis and thus improves stress tolerance.

### Regulation of *Lsi1* and *Lsi2* genes is responsible for Si accumulation in the shoot under osmotic stress and concomitant K deficiency

Like other elements, plants need transporters for uptake and translocation of Si from soil to different part of plant tissues (Ma and Yamaji, 2015). In barley, *HvLsi1* and *HvLsi2* transporters participate in Si uptake from external soil solution which is later released to the stele (Chiba et al., 2009; Ma and Yamaji, 2015). Recently, *HvLsi6* transporter was also identified for Si uptake in root tip and xylem unloading of Si in the leaf (Yamaji et al., 2012). In the present study, Si supply did not influence the K uptake (data not shown). However, Si concentrations increased in both root and shoot organs irrespective of the K level in the solution (Figures 2A,B). The accumulation of Si was more pronounced in the leaf of osmotic-stressed plants when Si was applied in higher concentration (Figure 2B). These results coincide with previous studies in rice plants (Chiba et al., 2009; Yamaji et al., 2015). The higher Si accumulation in the shoot was supported by the expression analysis of the genes involved in Si transport in barley showing a positive correlation with shoot Si concentration (Figures 4A,B). The positive correlation between *HvLsi1* and *HvLsi2* genes and Si concentration in the shoot was supported by the fact that only the induction of *HvLsi1* and *HvLsi2* genes was responsible for Si accumulation in barley leaf under osmotic stress and concomitant K deficiency (Figures 4A,B). Correlation analysis between Si concentrations in leaf and the expression level of *HvLsi6* was positive under control and K- deficient conditions. However, this correlation was negative when plants were exposed to osmotic stress (Figure 4C) indicating that *HvLsi6* did not contribute to Si accumulation in leaf under osmotic stress condition. Interestingly, under ample K supply, the expression of all three genes and Si concentration in shoot was strongly negative under both control and osmotic stress (Figures 5A–C), indicating the fact that induction of *HvLsi1* and *HvLsi2* genes is responsible for Si accumulation in leaf of barley only under osmotic stress and concomitant K deficiency. Yamaji and Ma (2007, 2011) showed that the expression of rice Si transporter *OsLsi1* and *OsLsi2* were down-regulated after imposing to drought stress for 4 h or treating with ABA for 9 h under –Si condition. In addition, Chiba et al. (2009) demonstrated that in barley and maize plants the expression of *HvLsi1* and *ZmLsi1* did not change by Si application while Mitani et al. (2009) showed that the expression of *HvLsi2* and *ZmLsi2* were down-regulated by Si supply. Down-regulation of *HvLsi6* also was observed in barley by Si supply (Yamaji et al., 2012). The reported variation in the expression of Si transporters in different studies mentioned above might be due to species dependent response (Lavinsky et al., 2016) or different ways of Si or stress treatments. For instance, as described by Ma and Yamaji (2015), rice contains two Casparian strips and both *OsLsi1* and *OsLsi2* cooperate for Si uptake and transport between symplastic and apoplastic pathways, while barley and maize comprises of only one Casparian strip and Si is first taken up from external solution by *HvLsi1* or *ZmLsi1* and then released to the stele by *HvLsi2* or *ZmLsi2*. Recently, Mitani-Ueno et al. (2016) illustrated that high Si accumulation in the shoot of rice plant is the cause for down-regulation of rice Si transporter *OsLsi1* and *OsLsi2*. In agreement with our finding, Kim et al. (2014) showed that the application of Si to cadmium (Cd) and copper (Cu) stressed plant increased the expression of *OsLsi1* and *OsLsi2* genes. Therefore, in the present study, the induction of barley Si transporters *HvLsi1* and *HvLsi2* were most likely triggered in response to osmotic stress and K deficiency to facilitate the transport of Si into the leaf for better performance under stress conditions.

### Si regulates ABA homeostasis under osmotic stress and concomitant K deficiency

Phytohormones play a unique role in plant response to biotic and abiotic stresses; this also holds true for nutrient deficiencies (Haeder and Beringer, 1981; Peuke et al., 2002). So far, there is no evidence indicating that Si interferes with phytohormone homeostasis (Mitani-Ueno et al., 2016). It has also been reported that both osmotic stress and K deficiency increased ABA levels (Hosseini et al., 2016). To evaluate the possible impact of Si on the stress hormone ABA, we measured ABA concentrations and examined the genes involved in ABA biosynthesis and catabolism pathways. Phytohormone analysis revealed that Si application did not change ABA levels in control conditions, whereas, under osmotic stress, Si supply affected the level of ABA (Figure 6). Under osmotic stress and K-limiting conditions, ABA concentrations decreased with increasing levels of Si in the medium, which coincided with a higher accumulation of the ABA degradation products PA and DPA (Figures 6A–C). These findings are in agreement with our previous work in barley, where drought tolerance of a genotype with higher K concentrations in the leaves was associated with higher turnover of ABA (Hosseini et al., 2016). In agreement with this, a study in rice showed that under salinity stress, the ABA level decreased with Si supply in a dose-dependent manner by regulating the genes involved in ABA biosynthesis (Kim et al., 2014). Kim et al. (2013) also showed in rice subjected to heavy metal stress that the ABA level initially decreased after 1 and 5 days of treatment with Si, however, with increasing duration of the stress, the ABA content increased. Our results show that Si accumulation reduced ABA level in a dose-dependent manner by induction of the ABA degradation pathway. This could be attributed to a transcriptional regulation of the expression of ABA biosynthesis or degradation genes. To answer the question, the transcript levels of genes mediating plastid and cytosolic pathways of ABA biosynthesis and degradation were determined. The results revealed a significant correlation between the expression level and the concentration of ABA (Figures 6, 7). Among the genes related to the plastid pathway, *HvZEP1* level strongly increased with increasing concentration of Si when plants were subjected to osmotic stress and concomitant K deficiency, while expression levels of *HvNCED1* and *HvNCED2* increased in a dose-dependent manner. The expression of *HvSDR1* and *HvSDR2*, i.e., genes related to the cytosolic pathway, also strongly increased under osmotic stress and K deficiency when Si concentrations were high. Determining the transcript levels of genes involved in ABA degradation, *HvABA8*′*OH-1, HvABA8*′*OH-2*, and *HvABA8*′*OH-3*, were found to be induced by Si supply and responded to osmotic stress and K deficiency (Figure 7). These results suggested that the higher turnover of ABA degradation products by Si may contribute to the overall decrease of the ABA concentration under osmotic stress and K deficiency.

Yamaji and Ma (2007, 2011) showed that a reduction in the expression level of *OsLsi1* gene under drought stress might be due to elevated ABA levels. These authors have demonstrated that the expression level of *OsLsi1* was strongly suppressed by ABA in a dose-dependent manner, resulting in lower Si uptake. Therefore, in the present study, decreased ABA levels by Si supply might be another reason for the observed induction of *HvLsi1* and *HvLsi2* gene expression under osmotic stress and concomitant K deficiency. However, further experiments are needed to investigate how ABA regulates the expression of Si transporter genes.

In addition to ABA, cytokinins (CKs) are plant hormones, which play a vital role in plant growth and development (Nishiyama et al., 2011). A number of findings demonstrated an antagonistic link between ABA and CKs that plays an important role in response to abiotic stresses (Nishiyama et al., 2011; Ha et al., 2012). Several studies have shown an interaction between CKs and different nutrients like nitrogen (Takei et al., 2004), phosphorus (Martin et al., 2000), sulfur (Maruyama-Nakashita et al., 2004), or K (Nam et al., 2012) that impacts on nutrient signaling in plants. Very recently, Markovich et al. (2017) have found an indirect link between Si and CKs. These authors have shown that a Si-mediated delay of senescence in Arabidopsis or sorghum was dependent on an increasing level of the active CK form isopentenyl adenine (iP), which finally led to a higher chlorophyll level in leaves and a lower expression level of the senescence marker gene *SAG12*. In close agreement with this result, the iP concentration was found to increase under osmotic stress and concomitant K deficiency in the presence of high Si concentrations (Figure 6D). This result indicates that under osmotic stress and K deficiency Si may interact with the CK pathway by increasing the iP level that alternatively reduces ABA level and thus delayed osmotic stress-induced leaf senescence.

### Silicon increased starch level and modulated glycolysis and TCA pathways

An important role for K in plant metabolism is evident from the fact that K deficiency affects the contents of primary and secondary metabolites (Amtmann et al., 2008). Several studies have also shown a close link between drought tolerance and carbohydrate metabolism in cereals (Seki et al., 2007; Armengaud et al., 2009; Hosseini et al., 2016). In our previous study in barley, we showed that sugars in particular insoluble starch, key metabolites of the TCA, 2-oxoglutarate and fumarate and glycolytic intermediates, glucose-6P, fructose-6P and 3-PGA, were much less affected by osmotic stress and K deficiency in high K retaining barley plant (Hosseini et al., 2016). In the present study, we focused on the same metabolic pathways and tested the additional effect of Si in combination with K deficiency and osmotic stress.

K deficiency and drought stress resulted in the accumulation of carbohydrates in leaves as replacement of osmotic molecules (Hermans et al., 2006). Also, sugar accumulation in leaves of K-deficient bean plants has been explained by a requirement for K in long-distance transport. Therefore, an impairment of phloem transport leads to decreased sugar levels in the roots (Cakmak et al., 1994). In the present study, the level of starch increased by Si application in spite of K level and Glu and Suc levels did not change in shoot in either treatment (Table 2). Compared to non-Si treated plants, Si supply reduced the level of Suc in shoot under osmotic stress and concomitant K deficiency (Table 2). In root, Suc level even increased upon Si application (Table 1). It was reported that accumulation of Suc in K-deprived plants is the reason for a decline in photosynthesis (Cakmak et al., 1994; Hermans et al., 2006). In rice plants, it was shown that the level of Suc, Fru, and Glu were reduced in *lsi1* mutant treated with Si at full grain burden stage compared to wild type plant (Detmann et al., 2012). Our results indicate that Si might prevent impairment of phloem transport of sugars in the roots and the decline of photosynthesis in the shoot (Armengaud et al., 2009) as supported by low sucrose level in the shoot. Also, to our knowledge, the present study is the first to show the effect of Si nutrition on increasing level of starch under osmotic stress and concomitant K deficiency. Starch is the main carbon pool in grains of cereals like barley and wheat comprising nearly 65–75% of the dry weight of the seed. It rarely accumulates in K-starved plants (Hermans et al., 2006). On the other hand, drought stress declines the photosynthetic rate and impairs carbohydrate metabolism and the level of starch (Mohammadkhani and Heidari, 2008; Farooq et al., 2009). Accumulation of starch and rearrangement of starch metabolism under osmotic stress have been reported (Thalmann et al., 2016; Zanella et al., 2016). Osmotic stress-induced accumulation of starch normally reduces cell water potential and maintains turgor pressure which is critical for plant survival and growth under stress conditions (Krasensky and Jonak, 2012). Starch also can act as a scavenger to protect the cell against oxidative stress by balancing the cellular redox (Couée et al., 2006; Miller et al., 2010). Therefore, our results suggest that Si enhances osmotic stress tolerance in barley plants in favor of starch accumulation in the shoot organs.

Our metabolite analysis of glycolytic intermediates and TCA cycle in root showed a high level of PEP, 3PGA, malate, fumarate, and citrate under osmotic stress and K deficiency when Si was applied in the medium (Table 1). A similar pattern was also observed for shoot metabolites, among glycolytic intermediates for sucrose-6-P, 3PGA, and PEP and among the TCA cycle for the levels of fumarate, citrate and isocitrate (Table 2). Armengaud et al. (2009) showed a decrease of organic acids in K-starved Arabidopsis leaves. We could not observe a reduction in organic acids in our study; even Si application elevated the level of glycolytic intermediates and TCA cycle metabolites under osmotic stress and concomitant K deficiency (Tables 1, 2). In agreement with our finding, Detmann et al. (2012) showed a strong correlation between Si concentration and levels of glycolytic intermediates isocitrate and 2-oxoglutarate in rice plants. These authors demonstrated that Si impact on amino acids remobilization by modulating the rate of 2-oxoglutarate. Taken together, based on our results we conclude that under osmotic stress condition, Si increases starch pool, which leads to an increase of carbon flux into glycolytic and TCA pathways that in turn results in a better performance of plant growth.

## Conclusion

**Figure 8:**
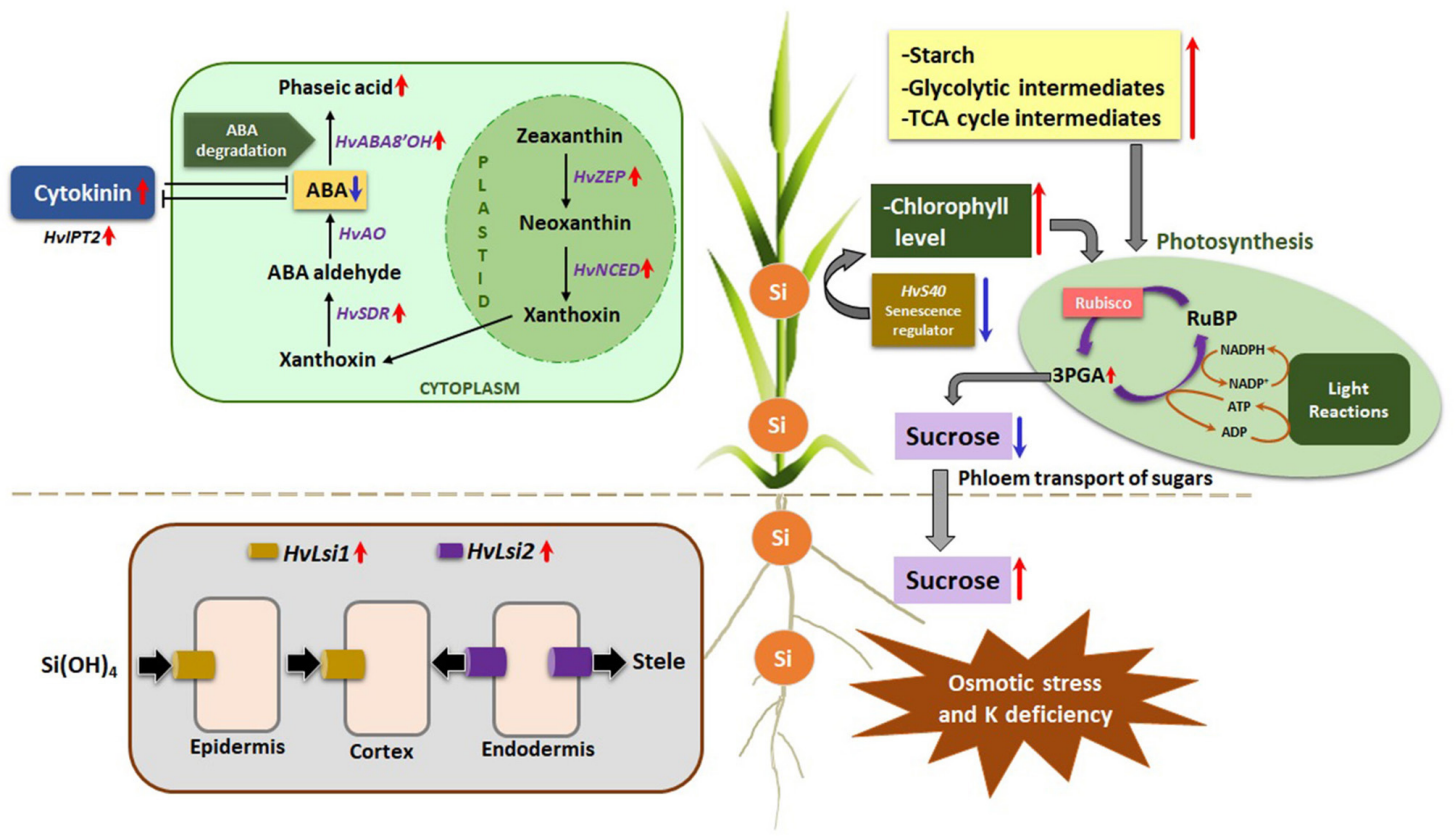
A Schematic model showing Silicon uptake by roots and its interaction with phytohormones and metabolic pathways under osmotic stress and concomitant K deficiency in Barley. Silicon uptake is mediated by upregulating the expression of *HvLsi1* and *HvLsi2* transporters in the roots under osmotic stress and concomitant K deficiency. Si is further translocated to the shoots where its accumulation delays the osmotic stress-induced leaf senescence through augmentation of chlorophyll level and downregulation of senescence marker gene *HvS40*. Si in the shoots interacts with metabolic pathways leading to an escalation of starch and consequently protecting cells from oxidative stress. Si also moderates the phloem transport of sugars from shoot to roots, thus increasing the sucrose levels in roots and subsequent increment of the carbon flux into glycolytic and TCA pathways. Another interesting aspect of higher silicon in shoots is its interaction with phytohormones that eventually leads to lower ABA levels, which may be attributed to the Si-mediated modulation of the transcriptional regulation of ABA biosynthesis and degradation pathway or its interaction leading to higher levels of active cytokinin iP. Considering all the probable interactions of Si, it may be emphasized that Si definitely plays a positive role in stress tolerance under K deficiency and other abiotic stress like osmotic stress. (Red arrows = upregulation, Blue arrows = downregulation).

Based on the results achieved in the present investigation, a schematic model is proposed to highlight the beneficial effects of Si under osmotic stress and concomitant K deficiency (Figure 8). (1) Osmotic stress and K deficiency induced the expression of Si transporters *HvLsi1* and *HvLsi2* in root which resulted in the accumulation of Si in the shoot. (2) The higher accumulation of Si in the shoot delayed osmotic stress-induced leaf senescence by increasing chlorophyll level and suppression of senescence marker gene *HvS40*. (3) Si also contributed to increased starch levels in shoot and Suc levels in root resulting in phloem transport of sugar to the root and an increase of carbon flux into glycolytic and TCA pathways. (4) Accumulation of Si by osmotic stress and concomitant K deficiency, leading to lower ABA levels in the shoot by modulating transcriptional regulation of ABA biosynthesis and degradation pathways and/or by increasing the levels of active cytokinin iP. Thus, our findings revealed a protective mechanism of Si during osmotic stress and K deficiency in barley and emphasize the importance of Si to effectively improve stress tolerance under K deficient condition in particular when additional abiotic stress like osmotic stress interferes.

## Author contributions

AM conducted the experiments and analyzed data. MH supervised the metabolite analysis. NA performed gene expression analysis. SH and JY designed the experiments and evaluated the data. AS and FJ supervised the hormone analysis. SH, JY, NA, and MH wrote the article.

### Conflict of interest statement

The authors declare that the research was conducted in the absence of any commercial or financial relationships that could be construed as a potential conflict of interest.
