# Supplementary material for: Induction of Barley Silicon Transporter HvLsi1 and HvLsi2, increased silicon concentration in the shoot and regulated Starch and ABA Homeostasis under Osmotic stress and Concomitant Potassium Deficiency
Source: Front Plant Sci. 2017 Aug 3;8:1359. doi: 10.3389/fpls.2017.01359 (PMC5541011; doi:10.3389/fpls.2017.01359)
Supplement: Supplementary file 1 [file Table1.DOCX]

Supplemental Table 1. List of primers used for qRT-PCR.

| **GENE** | **ACCESSION NO.** | **FORWARD PRIMER** | **REVERSE PRIMER** | **AMPLICON SIZE (BP)** |
| --- | --- | --- | --- | --- |
| ***HvLsi1*** | AB447482 | 5’-TAGAGCGGTTGGTGAGTTGG-3’ | 5’-CCAGGAAGTAGAGCCAGAGG-3’ | 161 |
| ***HvLsi2*** | AB447483 | 5’-CTGGATGATGGCGGTGTTC-3’ | 5’-ATGGTGGAGAAGAGCAGGC-3’ | 162 |
| ***HvLsi6*** | AB447484 | 5’-GCAGGGTTAGCAGTTGGTT-3’ | 5’-CGAAGCGGATGTAGGTGTAG-3’ | 196 |
| ***HvLhcb1*** | AB500090 | 5’-AGCCCCACTGTTAGCGAG-3’ | 5’-CGAAGGACGAGGAGGAGAG-3’ | 94 |
| ***HvRbcS*** | U43493 | 5’-GGTTGGGTTCATCTTCCGTG-3’ | 5’-GACCTCCTCCACCTCGTT-3’ | 130 |
| ***HvRbcL*** | LN626641 | 5’-GCAGCATTCCGAGTAAGTCC-3’ | 5’-GGTAAGTCCATCAGTCCAAACA-3’ | 114 |
| ***HvZEP1*** | MLOC_64943 | 5’-GCTATGGGTGGAGAGTGGT-3’ | 5’-AAGGGCAACGACAACGAAG-3’ | 152 |
| ***HvNCED1*** | DQ145930 | 5’-CGTCGGAGATGATGTGGGT-3’ | 5’-CCGTGTCGTTGAAGATGGAG-3’ | 138 |
| ***HvNCED2*** | DQ145931 | 5’-GGTGCTCGACAAGGAGAAGA-3’ | 5’-AGAGGTGGAAGCAGAAGCA-3’ | 110 |
| ***HvSDR1*** | MLOC_25903 | 5’-CAGTTGGATTCGGAAGAGGACA-3’ | 5’-CACAGCAAGATTAGCACGCA-3’ | 75 |
| ***HvSDR2*** | AK357628 | 5’-GCCATCTCGCCCAACTACA-3’ | 5’-CCTCCATCTCGTTGATGTCCC-3’ | 121 |
| ***HvAO2*** | MLOC_6141 | 5’-AGTGTGGCTGTTGGATTTCTC-3’ | 5’-GCGTGAAGACAATGGAAGGT-3’ | 138 |
| ***HvAO3*** | MLOC_56074 | 5’-CTACCCGAAATCACGCAGAA-3’ | 5’-CACCAAAGCCACCTCCAAC-3’ | 97 |
| ***HvABA8OH1*** | DQ145932 | 5’-CATTGCTGTCCATCTTCGGG-3’ | 5’-TTCACCGGCATCGAGTTGT-3’ | 100 |
| ***HvABA8OH2*** | DQ145933 | 5’-CAGGTGGGAGGTTGTTGGA-3’ | 5’-GTCATCTTCATCAGTCGGGC-3’ | 139 |
| ***HvABA8OH3*** | AK357473 | 5’-ATGGAGGAGCACTGGGAC-3’ | 5’-CTGCGATTCCTTGGCGTC-3’ | 105 |
| ***HvTSA*** | MLOC_12252 | 5’-GCGGAGAGGATGAAGGAGA-3’ | 5’-TTCCAAAGCCAACAGCCAC-3’ | 163 |
| ***HvTDC*** | AB162961 | 5’-ATCAAGGCAAGGGGAGACA-3’ | 5’-CCAACAGCAAAACGAAGCAC-3’ | 140 |
| ***HvIPT2*** | MLOC_76403 | 5’-ACCCCTCTAAAATCTGCCCTC-3’ | 5’-GACCACCACCACTGCCTT-3’ | 147 |
| ***HvNPR1*** | AM050559 | 5’-ACGCTGTTCAAATGTGCTGG-3’ | 5’-AACTCCTCTTTGTCCTCGCT-3’ | 147 |
| ***HvPR1a*** | X74939 | 5’-GGCGGCGTCTTCATCACCT-3’ | 5’-AGGAACGAGGGACTACTGGACTATG-3’ | 119 |
| ***HvPR1b*** | X74940 | 5’-GCCCAGAACTACGCCAACCA-3’ | 5’-CGTCCGATGCCTTCCAGTCC-3’ | 115 |
| ***HvPR2*** | AF515785 | 5’-GCATAGCATTTGAGCACCAGA-3’ | 5’-CACACCCGTAGGAACAGCA-3’ | 117 |
| ***HvPR5*** | AJ276225 | 5’-CGCAGAGCAACAACAGTAAAG-3’ | 5’-AAACAGCAAGGAGGAGGAAGA-3’ | 80 |
| ***HvS40*** | MLOC_7793 | 5’-TGTACCAACGACGCGAAGAG-3’ | 5’-AGGTTCCTGATATTGCGGAGG-3’ | 149 |
