# Supplementary material for: Induction of Barley Silicon Transporter HvLsi1 and HvLsi2, increased silicon concentration in the shoot and regulated Starch and ABA Homeostasis under Osmotic stress and Concomitant Potassium Deficiency
Source: Front Plant Sci. 2017 Aug 3;8:1359. doi: 10.3389/fpls.2017.01359 (PMC5541011; doi:10.3389/fpls.2017.01359)
Supplement: Supplementary file 2 [file DataSheet1.DOCX]

**A**

**Supplemental Figure 1: Influence of Si supply on concentrations and expression levels of genes involved in SA, IAA, GA3 and ACC biosynthesis pathways in fully expanded leaves of barley under osmotic stress.** A) heat map representation of genes involved in SA, IAA, GA and auxin biosynthesis/signalling pathways, B) SA, C) IAA, D) GA3 and E) ACC concentrations in fully expanded leaves of barley. Plants were grown in hydroponic culture under low K (0.05 mM), high K (1 mM) and two concentrations of Si (0.5 and 1 mM). Fully expanded leaves from 25-days old plants were harvested 5 days after imposition of osmotic stress. Osmotic stress applied by polyethylene glycol (PEG 6000, 19% (w/v)) to achieve osmotic stress levels of approximately -0.5 MPa. Purple color indicates a decrease and orange indicates an increase in gene expression levels of different hormonal pathways. Different shades of purple and orange express the extent of the change according to the color bar provided (log10). White indicates no change. Bars indicate means ± SE. Different letters denote significant differences according to SNK test (p < 0.05; n = 3).
